# Growth-Phase-Dependent Modulation of Quorum Sensing and Virulence Factors in *Pseudomonas aeruginosa* ATCC 27853 by Sub-MICs of Antibiotics

**DOI:** 10.3390/antibiotics14070731

**Published:** 2025-07-21

**Authors:** Ahmed Noby Amer, Nancy Attia, Daniel Baecker, Rasha Emad Mansour, Ingy El-Soudany

**Affiliations:** 1 Microbiology and Immunology Department, Faculty of Pharmacy and Drug Manufacturing, Pharos University in Alexandria, Alexandria 21648, Egypt; 2 Microbiology Department, Medical Research Institute, Alexandria University, Alexandria 26571, Egypt; 3 Department of Pharmaceutical and Medicinal Chemistry, Institute of Pharmacy, Freie Universität Berlin, Königin-Luise-Straße 2+4, 14195 Berlin, Germany; 4 Alexandria Main University Hospital, Alexandria 21526, Egypt

**Keywords:** quorum sensing, sub-MIC, growth phases, *Pseudomonas aeruginosa*, ciprofloxacin, amikacin, azithromycin, ceftazidime, meropenem

## Abstract

**Background**: Antibiotics at sub-inhibitory concentrations can rewire bacterial regulatory networks, impacting virulence. **Objective**: The way that exposure to selected antibiotics (ciprofloxacin, amikacin, azithromycin, ceftazidime, and meropenem) below their minimum inhibitory concentration (sub-MIC) modulates the physiology of *Pseudomonas aeruginosa* is examined in this study using growth-phase-resolved analysis. **Methods**: Standard *P. aeruginosa* strain cultures were exposed to ¼ and ½ MIC to determine the growth kinetics under antibiotic stress. The study measured protease and pyocyanin production and the expression level of important quorum sensing and virulence genes (*lasI*/*R*, *rhlI*/*R*, *pqsR*/*A*, and *phzA*) at different growth phases. **Results**: Meropenem produced the most noticeable growth suppression at ½ MIC. Sub-MIC antibiotics did not completely stop growth, but caused distinct, dose-dependent changes. Azithromycin eliminated protease activity in all phases and had a biphasic effect on pyocyanin. Ciprofloxacin consistently inhibited both pyocyanin and protease in all phases. The effects of amikacin varied by phase and dose, while β-lactams markedly increased pyocyanin production during the log phase. In contrast to the plateau phase, when expression was often downregulated or unchanged, most quorum-sensing- and virulence-associated genes showed significant upregulation during the death phase under sub-MIC exposure. **Conclusions**: These findings indicate that sub-MIC antibiotics act as biochemical signal modulators, preserving stress-adapted sub-populations that, in late growth phases, activate quorum sensing and stress tolerance pathways.

## 1. Introduction

Quorum sensing (QS) is a key regulator for the expression of *Pseudomonas aeruginosa* (*P. aeruginosa*) virulence factors, such as pyocyanin, rhamnolipid, elastase, protease, hemolysin, twitching and swarming motilities, as well as biofilm formation. QS presents an intercellular signaling pathway in bacteria that produces chemical signals, namely, autoinducers for cell-to-cell communication. When a quorum reaches a critical level, it induces the expression of genes encoding particular virulence factors of the bacteria [1].

The three QS systems in *P. aeruginosa*, *las*, *rhl*, and *pqs*, form an interdependent complex regulatory network that regulates the expression of many genes, where two systems use acyl-homoserine lactone (AHL) signals and the third system involves quinolone signals [2]. The *las* system is positioned at the top of the hierarchy of the QS system. It utilizes the signaling molecule 3-oxodecanoyl homoserine lactone (3OC12-HSL; synthesized by AHL synthase enzyme, *LasI*) and the regulator *LasR* to activate genes, such as *lasB* (elastase), and to influence other QS systems in an indirect manner. Similarly, the *rhl* system signaling molecule is butanoyl homoserine lactone (C4-HSL; synthesized by AHL synthase enzyme, *rhlI*) and the regulator is *rhlR*. The *rhl* system is controlled by the *las* system and regulates gene expression of some virulence factors, such as the biosurfactant rhamnolipid and the *phz* operon that is involved in pyocyanin production, contributing to oxidative stress and tissue damage. The *Pqs* system is driven through the signal 4-hydroxy-2-heptylquinoline (HHQ), which interacts with *pqsR* as the regulator. It coordinates with the *las* and *rhl* systems for influencing virulence, biofilm components, and other genes. In return, *pqs* also modulates the production and activity of the other QS systems. Collectively, these systems coordinate bacterial group behaviors, with complex feedback and regulation mechanisms that can vary among strains and environmental conditions [3].

Bacterial infections are mainly treated with antibiotics; however, the seriously increased emergence of resistance highlights the need for alternative therapeutic strategies. Antivirulence therapy provides a promising approach through targeting bacterial virulence—giving the upper hand to the immune system for resolving the infection—instead of applying selection pressure that leads to acquiring multidrug resistance [4]. Therefore, targeting and inhibiting QS pathways can impair the pathogenicity of *P. aeruginosa* and offers a potential antivirulence therapeutic strategy against its infections [1].

In this context, many antibiotics were studied for their efficacy in inhibiting bacterial virulence, where sub-minimum inhibitory concentrations (sub-MICs) of the antibiotics can greatly influence the bacterial transcription profile. It may also affect the QS signaling pathways more than affecting its growth as bacteriostatic or bactericidal. Hence, the production of some virulence factors may be upregulated or downregulated in the presence of the sub-MICs of antibiotics [4,5,6].

Macrolides, such as azithromycin (AZT), are well known to inhibit virulence factors of various bacteria, especially *P. aeruginosa*. Moreover, fluoroquinolones, like norfloxacin and ciprofloxacin (CIP), aminoglycosides, including amikacin (AMK) and streptomycin, and β-lactams (e.g., ceftazidime (CTZ), cefotaxime, and meropenem (MER)) were also studied for altering bacterial virulence during the late growth phases [5,6,7].

Accordingly, this study aimed to analyze the effect of sub-MICs of different antibiotic families during different growth phases on some virulence factors (phenotypically) and QS genes’ expression of *P. aeruginosa*. In particular, the standardized strain ATCC 27853 was used due to its good characterization, allowing for experimental reproducibility. To our best knowledge, this is the first study to elucidate the phase-dependent modulation of QS, thereby illustrating the variations in virulence phenotypically and genotypically in response to sub-MICs of antibiotics.

## 2. Results

In our study, the sub-MICs (¼ MIC and ½ MIC) for *P. aeruginosa* (ATCC 27853) were calculated from the MICs against the selected antibiotics, where the MIC of AZT was 64 μg/mL, CIP was 0.125 μg/mL, AMK was 2 μg/mL, MER was 0.25 μg/mL, and CTZ was 0.5 μg/mL.

### 2.1. Growth Curve Analysis

**Figure 1:**
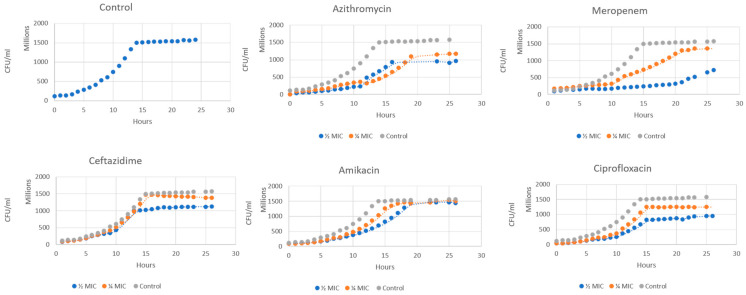
Growth curves of *P. aeruginosa* in the presence of antibiotics: azithromycin, meropenem, ceftazidime, amikacin, and ciprofloxacin, at the concentrations of 0 (●) (control), ¼ MIC (●), and ½ MIC (●) for 24 h. Dotted lines indicate phase transitions (log → plateau → death).

The growth kinetics of *P. aeruginosa* exhibited clear dose-dependent patterns in response to treatment with sub-MICs of different antibiotic classes (Figure 1). The control demonstrated a standard sigmoidal growth, reaching the plateau after nearly 15 h. Notably, none of the tested sub-MICs completely inhibited bacterial growth, but rather altered the growth kinetics and final culture density.

The most notable dose-dependent inhibition of culture growth was seen in MER. Bacterial growth was significantly suppressed at ½ MIC. However, an intermediate growth was permitted at ¼ MIC. This suggests that even at sub-MICs, there was substantial antibacterial action. At both tested doses, AZT and AMK showed considerable growth inhibition. Similar trajectories with partial growth suppression were seen in the growth curves for these antibiotics. The growth inhibition profiles of CIP and CTZ were comparable. Compared to the control, both antibiotics reduced the final bacterial density by about 25–30% at ½ MIC.

### 2.2. Pyocyanin Production

**Figure 2:**
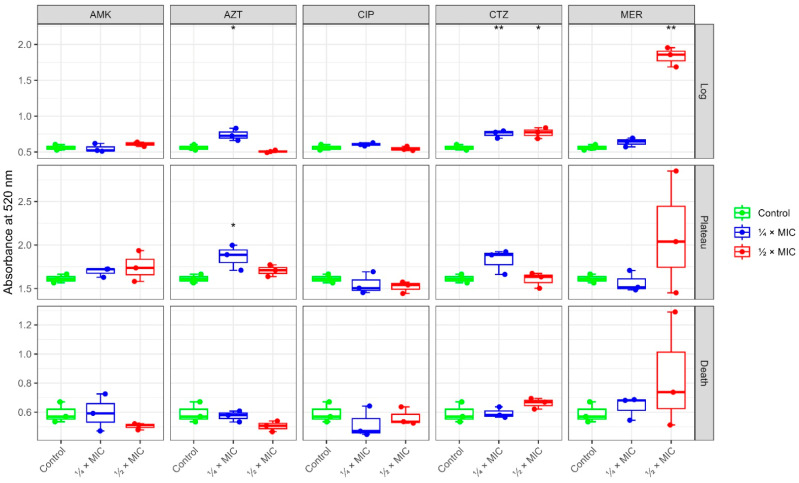
Effect of the used antibiotic panel in sub-MIC doses on the pyocyanin production measured at 520 nm in *P. aeruginosa*. CTZ: ceftazidime; AMK: amikacin; AZT: azithromycin; MER: meropenem; CIP: ciprofloxacin. Statistical analysis was done by analysis of variance (ANOVA) followed by Tukey’s Honest Significant Difference (HSD) test for pairwise comparisons. The asterisk * refers to statistical significance compared to the control at an alpha level < 0.05 and ** <0.01.

Pyocyanin (5-methyl-1(5*H*)-phenazinone) is a blue-green pigment that is found in *P. aeruginosa*. It acts as a virulence factor for the formation of reactive oxygen species (ROS). It is, therefore, suitable as a phenotypic marker for the response of *P. aeruginosa* to treatment with antibiotics. At both ¼ MIC and ½ MIC, the antibiotic CTZ caused a significant increase in pyocyanin production of *P. aeruginosa* during the log phase, while showing a little change in the plateau and death phases. MER affected pyocyanin production in a dose-dependent manner, enhancing its production, especially in the log phase, where the rise was significant with ½ MIC, while in the plateau and death phases no significant changes were observed. AZT exhibited a biphasic effect on the production of pyocyanin, where at low sub-MIC (¼ MIC), it promoted pigment synthesis. This was represented by a significant increase in pigment production at log and plateau levels. Higher sub-MIC levels suppressed the pigment production, with a slight decrease at the log and death phases. As for AMK and CIP, both showed relatively muted effects. Applying ¼ MIC of AMK and CIP generally hovered around control levels, and at ½ MIC both tended toward slight suppression, especially in the death phase, more prominently observed with CIP (Figure 2 and Table A1).

### 2.3. Protease Activity

**Figure 3:**
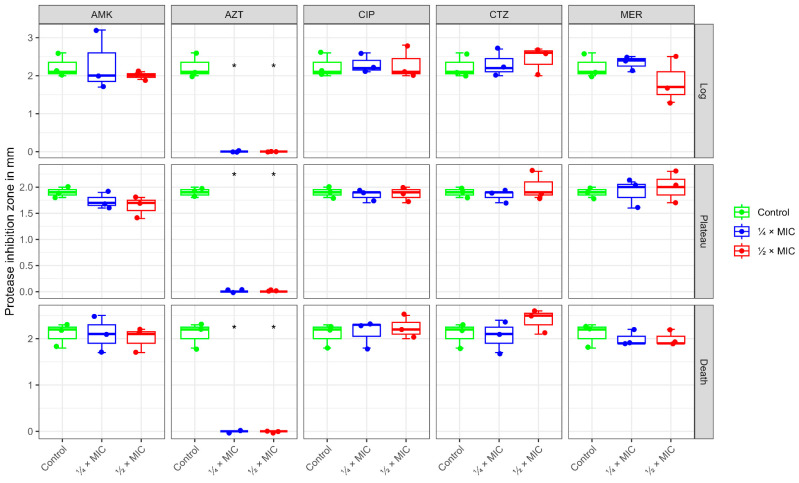
Effect of the used antibiotic panel in sub-MIC doses on the protease production in *P. aeruginosa*. CTZ: ceftazidime; AMK: amikacin; AZT: azithromycin; MER: meropenem; CIP: ciprofloxacin. Statistical analysis was done by ANOVA followed by Tukey’s HSD test for pairwise comparisons. The asterisk * refers to statistical significance compared to the control at an alpha level < 0.05.

*P. aeruginosa* produces several proteolytic enzymes (proteases). These contribute to the virulence of *P. aeruginosa*. Thus, addressing the effect of treatment with antibiotics on the activity of such proteases is another parameter to assess the impact of the drugs. At sub-MIC levels (¼ MIC and ½ MIC), AZT completely abolished the protease production of *P. aeruginosa* in all growth phases (log, plateau, and death), whereas CTZ had no significant impact at either tested concentration, but still a dose-dependent increase in log and death phases was observed. AMK exhibited inhibition of protease activity in all growth phases at both concentrations except for ¼ MIC at log phase, which showed an increased activity. Meanwhile, MER showed a different pattern. At ¼ MIC, an initial increase in protease activity at log phase, followed by gradual inhibition along the other phases, was observed. On the other hand, ½ MIC at log phase showed inhibition followed by a slight increase during the plateau. CIP produced a more complex, phase-specific response. Elevation of protease activity occurred in the log phase, suppressing it during the plateau at both doses, and modestly increasing it in the death phase at high doses, indicating neither a purely inhibitory nor consistently dose-dependent effect (Figure 3 and Table A2).

**Figure 4:**
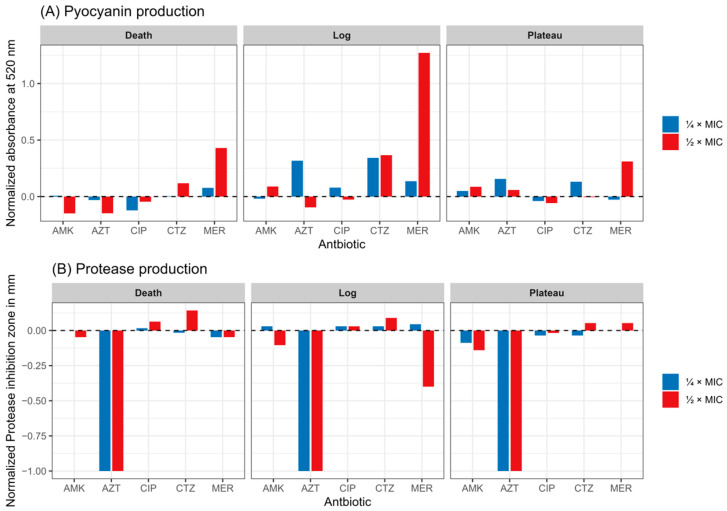
Pyocyanin (**A**) and protease (**B**) production after normalization to the control in each growth phase showing the effects of the five tested antibiotics at two sub-MICs (¼ MIC depicted in blue and ½ MIC displayed in red) on *P. aeruginosa* virulence.

To sum up the phenotypic trends presented in our study, the effect of antibiotics at sub-MIC revealed that CIP consistently inhibited protease and pyocyanin production across all phases. AMK effects varied according to the dose and growth phases. β-lactams (CTZ and MER) showed phase-dependent modulation of the virulence factors. An increase in pyocyanin production with β-lactams during both log and stationary phases was observed, while a slight decrease in protease production at both log and death phases occurred. As for AZT, the results showed a significant inhibition of protease activity across all phases. For pyocyanin production, AZT had a significant increase in both log and plateau phases, eventually suppressing pyocyanin production at the death phase (Figure 4).

### 2.4. Gene Expression Profile

**Figure 5:**
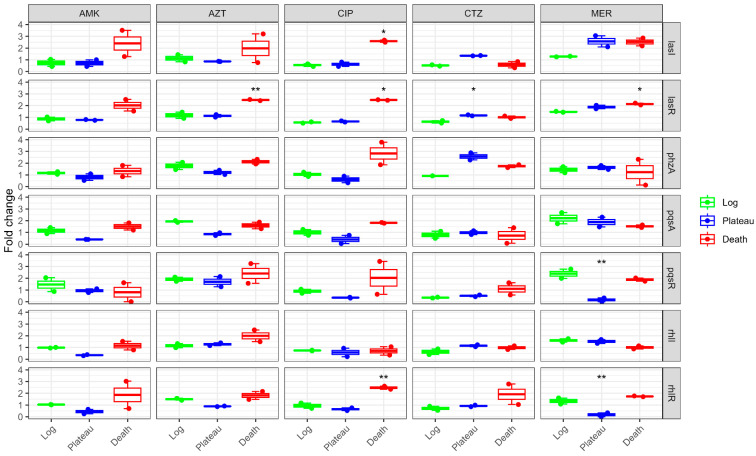
The effect of tested antibiotics at their sub-MICs on the expression of several virulence genes at different growth phases. CTZ: ceftazidime; AMK: amikacin; AZT: azithromycin; MER: meropenem; CIP: ciprofloxacin. Statistical analysis was done by ANOVA followed by Tukey’s HSD test for pairwise comparisons. The asterisk * refers to statistical significance compared to the control at an alpha level < 0.05 and ** <0.01.

Studying the effect of sub-MICs (at both ¼ MIC and ½ MIC) showed phase-dependent effects. In the log phase, AMK responded differently, exhibiting downregulation of the *las* system genes and upregulation of the *phzA* and *pqs* genes. During the plateau phase, all QS genes were downregulated in contrast to the death phase, which revealed upregulation. For the effect of sub-MICs of MER, all QS genes were upregulated in all growth phases except for *pqsR* and *rhlR* genes, which showed downregulation with statistical significance. The *phzA* gene expression was upregulated in all phases under the effect of CTZ sub-MICs. On the other hand, the remaining QS genes were downregulated in the log phase. The plateau phase showed overexpression of *las* system genes and under-expression of *pqs* genes. The death phase did not result in a uniform pattern of expression. Sub-MICs of AZT caused an overexpression in all QS genes in both log and death phases, without displaying a uniform pattern of expression in the plateau phase. Sub-MICs of CIP tended to downregulate all QS genes in both log and plateau phases and upregulate almost all genes in the death phase, with statistical significance observed with *las* and *rhl* genes (Figure 5 and Table A3).

**Figure 6:**
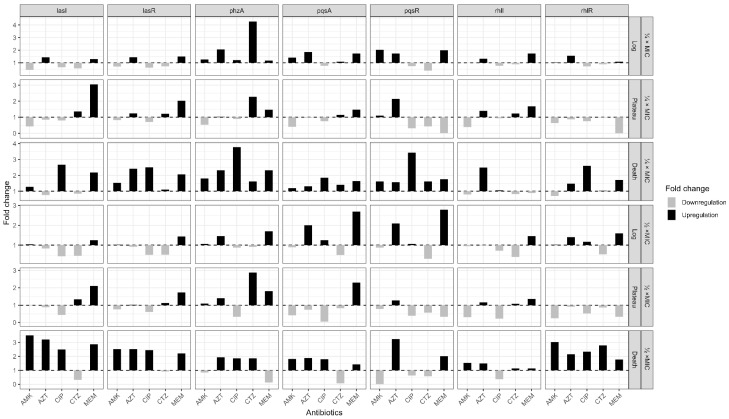
Fold change in gene expression of 7 virulence genes for 5 tested antibiotics in *P. aeruginosa*.

When having a deeper look at the effect of each sub-MIC on the expression of multiple virulence genes at different growth phases of *P. aeruginosa*, it was noticed that most of the studied genes were overexpressed during the death phase in both sub-MICs of the vast majority of the antibiotics. Concurrently, the plateau phase showed a trend of under-expression of the genes, which was more obvious in ½ MIC than in ¼ MIC. On the contrary, the log phase did not reveal a uniform pattern of gene expression. Low and high doses of antibiotics at sub-MICs affected the expression differently. The gene *lasI*/*lasR* in the log phase was overexpressed with ¼ MIC AZT, but ½ MIC under-expressed the *lasI* gene. Moreover, ¼ MIC AMK moderately repressed *lasI*/*lasR* and *rhlI*/*rhlR* in both log and plateau phases. This effect was less evident at ½ MIC (Figure 6).

## 3. Discussion

Mapping QS dynamics continuously across different growth phases under the stress of sub-MIC antibiotics from different classes fills a critical gap in understanding the pathophysiology of *P. aeruginosa*. QS autoinducers, such as AHLs and *pqs*, regulate the timed expression of virulence factors, such as biofilm matrix, proteases, and pyocyanin, and coordinate population-level behaviors [8,9]. Previous research on sub-MIC antibiotics has evaluated virulence outputs at discrete time points (e.g., mid-log or late stationary) [6,10], but it has not demonstrated how signal production and regulatory circuits evolve throughout the entire growth cycle. By profiling QS activity in real time under defined fractions of MIC for the common antibiotics CIP, MER, CTZ, AZT, and AMK, our work revealed the phase-dependent modulation of QS early repression, intermediate adaption, and late-phase rebound, shedding light on the shifts in virulence expression and antibiotic tolerance. This perspective is essential for explaining the correlation between antibiotic stress and QS regulation during infection.

Exposure of *P. aeruginosa* to ¼ MIC and ½ MIC levels of various antibiotics produced a clear, dose-dependent growth modulation. In our experiments, MER imposed the strongest suppression. At ½ MIC, the cultures showed markedly reduced growth, while CIP and CTZ sub-MICs gave the least pronounced effect on the growth curve. Mojsoska et al. found that β-lactam (CTZ and MER) sub-MICs produced stalled growth, longer lag/exponential phases, and lower plateau optical densities (ODs) [11]. It was also noticed that even very low MER doses (^1^/_16_ MIC) caused an increased division time [12]. Macrolides such as AZT are classically antivirulent, but sub-MICs can still affect growth. AZT produced a growth inhibition at both sub-MICs, with a delayed log phase and a depressed plateau. Similarly, Aleanizy et al. reported reduced growth with a nearly 70% reduction in the final OD of *P. aeruginosa* growth with AZT [6].

Importantly, none of the sub-MIC treatments completely prevented growth. All cells eventually reached the stationary phase at a lower density. These patterns align with previously published reports that low antibiotic levels rarely inhibit *P. aeruginosa* cultures but can only slow growth [13]. Similar to our findings, Yasir et al. have shown that CIP caused dose-dependent suppression of growth [14]. On the contrary, the dose-dependent growth suppression was not proved by Abu-Sini et al., who found that ¼ MIC of CIP or AMK had no significant impact on *P. aeruginosa* growth over several hours, whereas ½ MIC caused a delayed inhibition after about 6 h [10].

In general, sub-MICs tend to act more as stressors than killers. Goh et al. previously found that sub-MIC antibiotics can reprogram bacterial gene expression rather than causing lethality [15]. Consistent with this, our results revealed that even potent drugs (MER and CIP) at ½ MIC slowed growth.

The findings of this investigation support the reported patterns in the study of sub-MIC antibiotics. For instance, Mojsoska et al. published that sub-MIC β-lactams increased pyocyanin- and biofilm-associated toxicity in clinical isolates [11], mirroring the pyocyanin exacerbation observed in our study, where CTZ sub-MICs and ½ MIC MER significantly increased pyocyanin production at the log phase, suggesting that sub-therapeutic levels might trigger an aggressive, early virulence response. On the other hand, another study investigated the inhibitory effect of sub-MICs of β-lactams, such as CTZ, cefepime, and imipenem, on QS systems of *P. aeruginosa* and virulence factors, such as protease and pyocyanin [4]. In agreement with Khan et al. [16], who emphasized that sub-MIC levels of aminoglycosides often suppress virulence by binding QS receptors, our results almost gave suppression in virulence with AMK sub-MICs.

Sub-MICs of some antibiotics reshaped QS circuits in a phase/dose-dependent manner. MER and AZT upregulated the genes *lasI*, *lasR*, and *phzA* during both log and death phases, suggesting stress-induced activation of the *las* and phenazine systems. This aligns with reports that sub-MIC β-lactams enhance *lasR* expression under cell wall stress [17,18].

Conversely, CIP repressed *lasI*/*lasR* during growth but induced *lasI*/*lasR* in death phases, reflecting SOS-mediated virulence gene activation [19]. AMK repression of *lasI* at low doses and induction at higher doses underscores the biphasic effects of aminoglycosides on QS. At low doses, the antibiotic may disrupt the membrane slightly, enough to interfere with QS. At higher doses, more substantial membrane perturbation may activate stress response pathways, leading to increased QS activity as a survival mechanism [20,21].

AZT sub-MIC altered QS genes in a dose-dependent way. Here, ¼ MIC AZT upregulated *lasI*/*lasR* in the log phase (1.4–1.5× control), but ½ MIC suppressed *lasI* (to ~0.8×). Correspondingly, pyocyanin rose 30% at ¼ MIC (log/plateau) but was suppressed at ½ MIC. Our findings indicate that AZT low doses may initially stress the cells, causing upregulation of QS, whereas higher doses inhibit protein synthesis and QS regulator production. Notably, the observed complete shutoff of protease is consistent with previous observations that sub-MIC macrolides broadly suppress secreted virulence. However, all QS genes showed an overexpression with AZT sub-MIC at log and death phases not consistent with the phenotypic repression. Previous studies found that AZT may not significantly change messenger ribonucleic acid (mRNA) expression of QS-related genes but can lower the expression of enzymes involved in AHL synthesis, leading to reduced QS signaling [22]. Post-transcriptional regulatory mechanisms or changes in membrane permeability and the flux of QS signal molecules may play a role in these effects [23,24].

CIP tended to repress QS regulators during exponential growth. Our study showed modest repression of the *las*, *rhl*, and *pqs* systems in both log and stationary phases, yet elicited pronounced induction of these QS regulators in the death phase. Also, a decrease in protease activity during the stationary/death phases was observed and, moreover, a notable reduction in pyocyanin production. Therefore, sub-MIC CIP generally downregulates QS and virulence in *P. aeruginosa*. Abu-Sini et al. found that CIP low doses caused a significant reduction of *lasI*/*lasR* genes’ expression and related virulence genes [10]. The explanation of this could be due to the triggering of deoxyribonucleic acid (DNA) stress and SOS responses that attenuate QS signaling. Consequently, the synthesis of the early log-phase QS autoinducer AHL is reduced, which negatively affects the production of virulence factors later on [5]. Induction of DNA damage and bacterial SOS responses provide a stressful condition, leading to adaptation via upregulation of alternative QS pathways in the late phase [25].

Some studies indicated that β-lactams and cephalosporins have anti-QS activity. Kumar et al. reported an inhibition of virulence factors’ production, especially of pyocyanin and biofilm formation by sub-MIC of CTZ (and related cephalosporins) [26,27]. In this study, the picture was more complex. At ¼ MIC, CTZ modestly repressed *las*/*rhl* systems in the log phase, with an occasional slight rebound increase in stationary and/or death phases. Despite this, we observed a significant increase in the pyocyanin *phzA* gene at the early log phase, opposite to findings published by Kumar et al. [27]. Thus, our data suggest that the CTZ sub-MIC effect may be phase-dependent. The regulation of pyocyanin production involves a complex interplay between the *lasI*/*lasR*, *rhlI*/*rhlR*, and *pqs* systems. Discrepancies observed with CTZ may be due to its differential impact on these interconnected systems, particularly the *pqs* system and stress-induced pathways involving *PqsE* [28,29]. The discrepancy may also be strain or assay-dependent. Different strains of *P. aeruginosa* and variations in experimental conditions can lead to varying responses to the antibiotic [4,30].

In contrast, MER sub-MICs tended to suppress protease despite strong induction of all QS systems’ expression (up to 3-fold in early phases). Similar findings were reported by Deshamukhya et al. [18], who reported an increase in *lasI*/*lasR* and *rhlI*/*rhlR* genes’ expression. However, imipenem showed an opposite effect in the aforementioned study, suggesting that the effect is related to the molecule and not class-associated [18]. This specificity aligns with recent structural evidence showing that MER selectively binds to penicillin-binding protein 3 (PBP3), inducing cell elongation and division defects even at sub-MICs. This suggests that different cell wall inhibitors can interact with QS circuits in complex ways. This was accompanied by increased pyocyanin synthesis in the log and death phases and only modest protease suppression, especially in the death phase. This behavior may reflect cell-wall stress of MER, which is also endorsed by the effects seen on the growth curve, changing its magnitude and shape. Even at low doses, MER seems to stimulate QS circuit activation (perhaps as a stress response), rather than its complete inhibition. We noted that MER increased *las*/*rhl* systems’ expression while reducing protease output, suggesting SOS-independent post-transcriptional regulatory mechanisms or alterations in metabolic flux [23].

Aminoglycosides such as AMK also tend to downregulate QS at sub-inhibitory levels during bacterial growth. Abu-Sini et al. reported that sub-MIC AMK decreased *lasI*/*lasR* expression and reduced biofilm-related virulence genes [10]. Our results showed that *lasI*/*lasR* and *rhlI*/*rhLR* were moderately inhibited by ¼ MIC AMK in both log and plateau phases. This effect was less evident at ½ MIC. These effects were endorsed by lowering protease activity also in log/plateau phases. Pyocyanin production was largely unchanged at ¼ MIC and only weakly suppressed at ½ MIC during the death phase. Meanwhile, nearly all the QS circuits showed a resurgence of the expression of the gene in the death phase. Aminoglycoside-mediated ribosomal misreading causes early diminished QS protein fidelity, however, sustained proteotoxic stress and energy demands may stimulate a compensatory upregulation of QS as cells progress toward decline [31].

All of these effects may be justified by the fact that sub-MIC antibiotics function as biochemical signals, reprogramming the regulatory networks at low antibiotic doses rather than killing cells [32]. Antibiotics at sub-MIC levels may function as signaling mediators that broadly affect transcription and interfere with autoinducer synthesis and QS receptor function [33]. For example, sub-MIC aminoglycosides are known to cause mistranslation and proteotoxic stress, which indirectly disrupts QS systems by shifting cellular resources and regulatory priorities [34,35], which may trigger compensatory QS induction. Sub-MIC fluoroquinolones induce the SOS response, which can downregulate early QS autoinducers and upregulate alternative QS pathways [19]. Macrolides at sub-MIC levels exhibit a dual role by partially inhibiting protein synthesis and reducing virulence factor secretion, while potentially upregulating QS genes post-transcriptionally [36]. CTZ, a cephalosporin, has been shown to influence QS in *P. aeruginosa*, potentially by altering the membrane. This alteration can impair the accumulation or efflux of QS molecules, such as AHLs, which are crucial for bacterial communication [37]. The antibiotic is repurposed as a QS inhibitor (QSI). As an example of this, AZT and ceftriaxone both significantly suppressed *P. aeruginosa* virulence factor expression without killing the bacteria [38].

Our study demonstrated that bacterial responses to sub-MIC antibiotics vary by growth phase due to physiological differences. The majority of antibiotics temporarily suppressed the expression of *lasI*/*lasR* and *pqsA* during early exponential development, indicating an initial metabolic slowdown. This could be attributed to active metabolism and division enhancing antibiotic uptake and triggering stress responses like DNA or membrane stress, as seen with fluroquinolones, which may transiently suppress QS [9,15,39]. However, β-lactam antibiotics and AZT at low doses can paradoxically upregulate *las* and *phzA* before higher sub-MICs shut them down [22,40]. With decreased *pqs* output during early log and peak synthesis in stationary/death phases, the *pqs* system in particular demonstrated growth phase differential regulation of anthranilate metabolism and signal generation, which is consistent with phase-specific virulence modulation [41]. Interestingly, our data revealed that sub-MIC antibiotics override this baseline regulation. Growth phase coupling is probably disrupted by MER (by PBP3 inhibition), AZT, and AMK (via ribosomal targeting), which causes premature *pqs* activation as a stress-adaptation tactic. This implies that *pqs* is decoupled from its inherent growth phase restrictions by antibiotic stress. During the stationary phase, many QS circuits adapt or partially rebound with CIP, which causes SOS-mediated *pqsA*/*phzA* induction during the latter stages [19,31]. Finally, late-phase cultures often exhibit renewed QS activation or strong stress-driven virulence gene expression [32]. This indicates active survival mechanisms, such as efflux pumps or error-prone polymerases, often leading to a rebound in QS gene expression. Our data extended these patterns by revealing that late-phase QS resurgence under sub-MIC stress correlates with virulence rebound (e.g., MER-induced pyocyanin), suggesting persistent pathogenicity despite growth suppression.

The noted overexpression of QS genes in the late growth phase can induce oxidative stress, leading to the production of ROS, such as superoxide and hydrogen peroxide. This, in turn, increases the mutation rates in non-growing cells by SOS response and stress response sigma factors, like RpoS (RNA polymerase Sigma). This can indeed promote the formation of persister cells, contributing to bacterial survival during the death phase [42,43]. Research indicates that β-lactam sub-MICs in *P. aeruginosa* accelerate genetic variation by upregulating mutagenic pathways. It is important to note that late-phase survivors under sub-MIC exposure are primed for adaptive mutations and resistance evolution [44].

In this study, clinical isolates were not included, through which the effect of antibiotics at sub-MICs on more phenotypically virulent strains can be assessed, which may exhibit varied responses due to diverse resistance mechanisms. Sub-MIC antibiotics may also affect biofilm formation and other virulence factors. Future studies should explore the effects on virulence factors, such as rhamnolipids, and genes related to biofilm formation across different growth phases.

In addition, using antibiotic sub-MICs would provide a selection pressure for more resistant traits; therefore, mechanisms of antibiotic tolerance and resistance should have been studied in parallel to virulence factors’ expression. Nevertheless, studying combination therapies at sub-MIC levels represents an important future direction to explore potential interactions. Our study provided insights into QS and virulence factor dynamics as well as stress responses through log, plateau, and death phases under sub-MICs of antibiotics, which could contribute to our understanding of tolerance and development of resistance [45]. The previous studies did not address adaptability at the molecular level in different growth phases. In this way, our time-resolved QS and virulence profiles usher in the precise windows in which sub-MIC antibiotics preserve a viable survivor pool and actively induce adaptive programs, which potentially can lead to resistance emergence.

## 4. Materials and Methods

### 4.1. Bacterial Strain

In our study, a standard *P. aeruginosa* strain (ATCC 27853), obtained from the Medical Research Institute, Alexandria University, was used. All experiments were conducted under controlled laboratory conditions, standard medium, 37 °C, and an aerobic environment. The influence of environmental factors, such as changes in pH value, oxygen content, or nutritional status, was, therefore, not a subject of the current investigations.

### 4.2. Determination of Minimum Inhibitory Concentration of Studied Antibiotics

The MIC for each antibiotic was assessed using the broth microdilution method according to the Clinical and Laboratory Standards Institute (CLSI) guidelines [46]. Each antibiotic was repeated three times. After incubation for 24 h, at 37 °C, the MIC was observed as the lowest antibiotic concentration showing no visible bacterial growth. The recorded sub-MICs, ½ MIC and ¼ MIC, were used for subsequent phenotypic and genotypic investigations.

### 4.3. Growth Curve Determination

Growth curves of *P. aeruginosa* were constructed by incubation of 1 × 10^5^ CFU/mL of the used standard strain with the selected antibiotics at 0, ¼ MIC, and ½ MIC at 37 °C for 24 h for each antibiotic sub-MIC. Triplicates were performed. The bacterial count was determined every two hours by measuring the OD using BD PhoenixSpec™, Phoenix, AZ, USA. The device measures the OD of the suspension and reports the results in terms of McFarland units, which was converted to bacterial count. The average count was then calculated and used to plot the growth curve.

### 4.4. Phenotypic Detection of Pyocyanin Production

The used strain was cultured alone and with the sub-MICs (at ¼ MIC and ½ MIC) in glycerol alanine minimal medium at 37 °C for 24 h [47]. Sampling was done at the middle point of each phase. For each growth curve, the log phase sampling range was between 4 and 8 h, while the plateau was between 14 h and 18 h, and death phase sampling was carried out after 24 h. The MER sampling protocol was different due to the marked change in the growth curve shape and trajectory. After each sampling, the samples from the same growth phase were diluted to equal ODs. The cells were removed through centrifugation. The pyocyanin was extracted from the supernatant with chloroform, then re-extracted using 0.2 M HCl. For pyocyanin quantitation, absorbance was measured spectrophotometrically at 520 nm. The untreated cultures were considered the positive control, whereas a negative control was applied for methodology validation using a sterile culture medium. For each of the used antibiotic concentrations and the control, the test was repeated three times.

### 4.5. Phenotypic Detection of Protease Activity

The test was performed according to İnat et al. [48], involving a semi-quantitative method for detecting protease activity, still permissible to detect significant shifts and, therefore, sufficient for the purpose of this study. The advantage of this protease plate assay lies in its simplicity, cost efficiency, and rapid screening capability. The strain was cultured in Mueller-Hinton broth (MHB) alone and with tested antibiotics at sub-MICs. Samples were taken at different points of the growth phase, as previously described, and diluted to equal ODs. After centrifugation, the supernatant was collected for the determination of protease activity. In 2% skimmed milk agar plates, 40 μL of each supernatant was inoculated in wells made by sterile cork-borer (of 7 mm diameter) and incubated for 24 h, at 37 °C. The produced halo zones around the wells were measured in mm. For each of the used antibiotic concentrations and the control, the test was repeated three times.

### 4.6. Quantitative Reverse Transcription PCR Investigating the Expression of QS Genes and a Pyocyanin Gene

The effect of sub-MICs on the expression of QS and virulence genes in *P. aeruginosa* ATCC 27853 was assessed by SYBR green RT-PCR. The untreated and treated bacteria with ¼ MIC and ½ MIC were propagated, and cells were collected as described in the middle of each growth phase. Cells were harvested via centrifugation, and the total RNA was extracted by employing the Fast HQ RNA Extraction Kit (iNtRON Biotechnology, Seongnam-si, Republic of Korea). The concentration and the purity for each ribonucleic acid (RNA) sample were determined using a Nano-Drop Spectrophotometer (Thomas Scientific, Swedesboro, NJ, USA).

Complementary DNA (cDNA) was synthesized using the TOPscripit TM cDNA Synthesis Kit (enzynomics, Daejeon, Republic of Korea) according to the manufacturer’s instructions. The cDNA was used as a template for expression level analysis by using SYBR Green Master Mix (Maxima SYBR Green/ROX qPCR Master Mix, Thermo Fisher Scientific, San Jose, CA, USA). The PCR reaction was conducted as follows: denaturation at 95 °C for 30 s, annealing for 30 s at the specific temperature for each primer used (Table A4), and extension at 72 °C for 30 s. The specificity of the primers was confirmed via blasting on the National Center for Biotechnology Information (NCBI) database [49]. Melting curve analysis was done immediately after the final amplification step by heating the samples at 95 °C for 60 s, cooling to 55 °C for 30 s, and heating to 95 °C for 30 s with continuous fluorescence recording. Melting curves were recorded by plotting fluorescence signal intensity versus temperature. The complete procedure included amplification, data acquisition, and analysis by Mxpro-mx3000 (Stratagene, La Jolla, CA, USA). The expression level for QS and virulence genes was determined, using *ropd* as a reference gene.

### 4.7. Statistical Analyses

Data were analyzed in R Studio (R 4.4.2). Normality of data was tested by the Shapiro–Wilk test. Quantitative data were summarized as mean and standard deviation (SD). ANOVA, followed by Tukey’s HSD test for pairwise comparisons, was used to test normally distributed quantitative data. A value of <0.05 for the *p*-value was considered statistically significant.

## 5. Conclusions and Future Directions

Sub-MIC antibiotics can drastically alter *P. aeruginosa* behavior by modulating QS and associated virulence gene expression. None of the tested sub-MICs completely prevented bacterial growth but they modulated the growth kinetics and final bacterial density. Phenotypically, CIP consistently inhibited pyocyanin production across all phases. Effects of AMK varied according to the dose and growth phases, while β-lactams (MER and CTZ) showed phase-dependent modulation of the virulence factors. AZT abolished protease activity across all phases, showing a biphasic effect on pyocyanin. At the genetic level, QS and virulence genes were significantly upregulated during the death phase under sub-MIC exposure, whereas expression was often downregulated or unchanged in the plateau phase. These data suggest that sub-MIC antibiotics function as biochemical signal modulators, reprogramming the regulatory networks. Sub-MIC antibiotics stress can preserve stress-adapted reservoirs, which, in late phases, could activate QS pathways. Further studies addressing the mechanisms of antibiotic tolerance and resistance in parallel to virulence factor expression are required to be studied across growth phases. Also, future research should be extended to clinical strains to validate the findings of the current study and enhance their clinical applicability. Direct quantification of QS signal molecules and key virulence factors, like elastase, is also required to validate transcriptional and phenotypic effects.

## Data Availability

Data are available upon request from the corresponding authors.

## Abbreviations

The following abbreviations are used in this manuscript:

AHL	Acyl-homoserine lactone
AMK	Amikacin
ANOVA	Analysis of variance
AZT	Azithromycin
cDNA	Complementary DNA
CFU	Colony-forming unit
CIP	Ciprofloxacin
CLSI	Clinical and Laboratory Standards Institute
CTZ	Ceftazidime
DNA	Deoxyribonucleic acid
HHQ	2-Heptylquinol
HSD	Honest significant difference
MER	Meropenem
MHB	Mueller-Hinton broth
MIC	Minimum inhibitory concentration
mRNA	Messenger RNA
NCBI	National Center for Biotechnology Information
OD	Optical density
PBS	Phosphate-buffered saline
PBP	Penicillin-binding protein
PCR	Polymerase chain reaction
qRT-PCR	Quantitative reverse transcription PCR
QS	Quorum sensing
QSI	QS inhibitor
RNA	Ribonucleic acid
ROS	Reactive oxygen species
SD	Standard deviation
sub-MIC	Sub-inhibitory concentration

## Appendix A

**Table A1:** Effects of the used antibiotic panel in sub-MIC doses on the pyocyanin production in *P. aeruginosa*.

Antibiotics	Growth Phase	Pyocyanin OD	Control	¼ MIC	½ MIC	*p*
**CTZ**	Log	Mean ± SD	0.561 ± 0.038 ^a,b^	0.753 ± 0.055 ^a^	0.767 ± 0.076 ^b^	0.009 *
	Plateau	Mean ± SD	1.612 ± 0.050	1.823 ± 0.142	1.603 ± 0.089	0.062
	Death	Mean ± SD	0.592 ± 0.071	0.594 ± 0.038	0.662 ± 0.037	0.241
**AMK**	Log	Mean ± SD	0.561 ± 0.038	0.551 ± 0.060	0.611 ± 0.031	0.284
	Plateau	Mean ± SD	1.612 ± 0.050	1.692 ± 0.055	1.751 ± 0.178	0.371
	Death	Mean ± SD	0.592 ± 0.071	0.596 ± 0.127	0.504 ± 0.023	0.382
**AZT**	Log	Mean ± SD	0.561 ± 0.038 ^b^	0.739 ± 0.086 ^a,b^	0.508 ± 0.017 ^a^	0.005 *
	Plateau	Mean ± SD	1.612 ± 0.050 ^a^	1.865 ± 0.146 ^a^	1.706 ± 0.067	0.049 *
	Death	Mean ± SD	0.592 ± 0.071	0.574 ± 0.038	0.504 ± 0.037	0.166
**MER**	Log	Mean ± SD	0.561 ± 0.038 ^b^	0.637 ± 0.061 ^a^	1.833 ± 0.136 ^a,b^	<0.001 *
	Plateau	Mean ± SD	1.612 ± 0.050	1.568 ± 0.121	2.113 ± 0.703	0.275
	Death	Mean ± SD	0.592 ± 0.071	0.637 ± 0.080	0.846 ± 0.400	0.432
**CIP**	Log	Mean ± SD	0.561 ± 0.038	0.605 ± 0.022	0.546 ± 0.031	0.124
	Plateau	Mean ± SD	1.612 ± 0.050	1.550 ± 0.126	1.519 ± 0.068	0.46
	Death	Mean ± SD	0.592 ± 0.071	0.520 ± 0.107	0.565 ± 0.062	0.582

CTZ: ceftazidime; AMK: amikacin; AZT: azithromycin; MER: meropenem; CIP: ciprofloxacin; SD: standard deviation. ANOVA followed by Tukey’s HSD test was used for pairwise comparisons. The asterisk * refers to statistical significance compared to the control at an alpha level < 0.05. Common superscript letters refer to significant pairwise comparisons between groups.

**Table A2:** Effects of the used antibiotic panel in sub-MIC doses on the protease production in *P. aeruginosa*.

Antibiotics	Growth Phase	Protease Inhibition Zone in mm	¼ MIC	½ MIC	Control	*p*
**CTZ**	Log	Mean ± SD	2.300 ± 0.361	2.433 ± 0.379	2.233 ± 0.321	0.788
	Plateau	Mean ± SD	1.833 ± 0.115	2.000 ± 0.265	1.900 ± 0.100	0.542
	Death	Mean ± SD	2.067 ± 0.351	2.400 ± 0.265	2.100 ± 0.265	0.377
**AMK**	Log	Mean ± SD	2.300 ± 0.794	2.000 ± 0.100	2.233 ± 0.321	0.751
	Plateau	Mean ± SD	1.733 ± 0.153	1.633 ± 0.208	1.900 ± 0.100	0.200
	Death	Mean ± SD	2.100 ± 0.400	2.000 ± 0.265	2.100 ± 0.265	0.906
**AZT**	Log	Mean ± SD	0.000 ± 0.000 ^a^	0.000 ± 0.000 ^b^	2.233 ± 0.32 ^a,b^	<0.001 *
	Plateau	Mean ± SD	0.000 ± 0.000 ^a^	0.000 ± 0.000 ^b^	1.900 ± 0.100 ^a,b^	<0.001 *
	Death	Mean ± SD	0.000 ± 0.000 ^a^	0.000 ± 0.000 ^b^	2.100 ± 0.265 ^a,b^	<0.001 *
**MER**	Log	Mean ± SD	2.333 ± 0.208	1.833 ± 0.611	2.233 ± 0.321	0.361
	Plateau	Mean ± SD	1.900 ± 0.265	2.000 ± 0.300	1.900 ± 0.100	0.842
	Death	Mean ± SD	2.000 ± 0.173	2.000 ± 0.173	2.100 ± 0.265	0.801
**CIP**	Log	Mean ± SD	2.300 ± 0.265	2.300 ± 0.436	2.233 ± 0.321	0.964
	Plateau	Mean ± SD	1.833 ± 0.115	1.867 ± 0.153	1.900 ± 0.100	0.813
	Death	Mean ± SD	2.133 ± 0.289	2.233 ± 0.252	2.100 ± 0.265	0.824

CTZ: ceftazidime; AMK: amikacin; AZT: azithromycin; MER: meropenem; CIP: ciprofloxacin; SD: standard deviation. ANOVA followed by Tukey’s HSD test was used for pairwise comparisons. The asterisk * refers to statistical significance compared to the control at an alpha level < 0.05. Common superscript letters refer to significant pairwise comparisons between groups.

**Table A3:** Effects of tested antibiotics at their sub-MIC on expression of multiple virulence genes at different growth phases.

Genes	Antibiotics	Fold Change	Log	Plateau	Death	*p*
***lasI***	CTZ	Mean ± SD	0.521 ± 0.073	1.352 ± 0.020	0.587 ± 0.376	0.056
	AZT	Mean ± SD	1.133 ± 0.426	0.865 ± 0.017	1.984 ± 1.726	0.584
	MER	Mean ± SD	1.279 ± 0.038	2.578 ± 0.672	2.523 ± 0.473	0.117
	CIP	Mean ± SD	0.548 ± 0.152 ^b^	0.622 ± 0.257 ^a^	2.586 ± 0.127 ^a,b^	0.003 *
	AMK	Mean ± SD	0.739 ± 0.425	0.720 ± 0.401	2.396 ± 1.579	0.286
***lasR***	CTZ	Mean ± SD	0.623 ± 0.142 ^a^	1.171 ± 0.059 ^a^	1.012 ± 0.141	0.042 *
	AZT	Mean ± SD	1.179 ± 0.363 ^b^	1.131 ± 0.149 ^a^	2.474 ± 0.068 ^a,b^	0.016 *
	MER	Mean ± SD	1.475 ± 0.054 ^a^	1.879 ± 0.207	2.136 ± 0.110 ^a^	0.039 *
	CIP	Mean ± SD	0.564 ± 0.073 ^b^	0.653 ± 0.071 ^a^	2.478 ± 0.035 ^a,b^	<0.001 *
	AMK	Mean ± SD	0.864 ± 0.228	0.784 ± 0.045	2.030 ± 0.700	0.102
***phzA***	CTZ	Mean ± SD	2.596 ± 2.370	2.576 ± 0.427	1.745 ± 0.171	0.799
	AZT	Mean ± SD	1.765 ± 0.432	1.215 ± 0.254	2.131 ± 0.281	0.148
	MER	Mean ± SD	1.442 ± 0.364	1.632 ± 0.238	1.232 ± 1.552	0.914
	CIP	Mean ± SD	1.048 ± 0.247	0.612 ± 0.396	2.825 ± 1.356	0.142
	AMK	Mean ± SD	1.163 ± 0.145	0.812 ± 0.393	1.328 ± 0.680	0.582
***pqsA***	CTZ	Mean ± SD	0.804 ± 0.415	0.982 ± 0.224	0.741 ± 0.938	0.921
	AZT	Mean ± SD	1.937 ± 0.100	0.857 ± 0.163	1.599 ± 0.396	0.051
	MER	Mean ± SD	2.216 ± 0.663	1.888 ± 0.586	1.527 ± 0.153	0.499
	CIP	Mean ± SD	1.008 ± 0.346	0.409 ± 0.497	1.822 ± 0.045	0.061
	AMK	Mean ± SD	1.156 ± 0.365	0.409 ± 0.006	1.508 ± 0.434	0.092
***pqsR***	CTZ	Mean ± SD	0.354 ± 0.053	0.505 ± 0.098	1.096 ± 0.732	0.323
	AZT	Mean ± SD	1.917 ± 0.248	1.709 ± 0.614	2.409 ± 1.188	0.691
	MER	Mean ± SD	2.386 ± 0.556 ^b^	0.166 ± 0.225 ^a,b^	1.887 ± 0.189 ^a^	0.018 *
	CIP	Mean ± SD	0.896 ± 0.232	0.348 ± 0.052	2.036 ± 1.978	0.433
	AMK	Mean ± SD	1.458 ± 0.826	0.944 ± 0.229	0.812 ± 1.141	0.734
***rhlI***	CTZ	Mean ± SD	0.641 ± 0.337	1.151 ± 0.113	0.985 ± 0.220	0.246
	AZT	Mean ± SD	1.159 ± 0.240	1.276 ± 0.168	1.996 ± 0.708	0.269
	MER	Mean ± SD	1.603 ± 0.204	1.516 ± 0.235	1.010 ± 0.180	0.117
	CIP	Mean ± SD	0.744 ± 0.035	0.578 ± 0.506	0.710 ± 0.501	0.916
	AMK	Mean ± SD	0.985 ± 0.041	0.349 ± 0.063	1.160 ± 0.519	0.143
***rhlR***	CTZ	Mean ± SD	0.720 ± 0.244	0.922 ± 0.090	1.916 ± 1.235	0.346
	AZT	Mean ± SD	1.489 ± 0.113	0.889 ± 0.035	1.816 ± 0.484	0.102
	MER	Mean ± SD	1.340 ± 0.363 ^a^	0.169 ± 0.236 ^a,b^	1.738 ± 0.047 ^b^	0.017 *
	CIP	Mean ± SD	0.945 ± 0.316 ^a^	0.642 ± 0.160 ^b^	2.474 ± 0.182 ^a,b^	0.008 *
	AMK	Mean ± SD	1.034 ± 0.013	0.442 ± 0.273	1.863 ± 1.644	0.438

CTZ: ceftazidime; AMK: amikacin; AZT: azithromycin; MER: meropenem; CIP: ciprofloxacin; SD: standard deviation. ANOVA followed by Tukey’s HSD test was used for pairwise comparisons. The asterisk * refers to statistical significance compared to the control at an alpha level < 0.05. Common superscript letters refer to significant pairwise comparisons between groups.

**Table A4:** List of primers used in our study and their annealing temperatures [49].

Primer Name	Primer Sequence	Annealing
*rpod F*	5′CGAACTGCTTGCCGACTT3′	53 °C
*rpod R*	5′GCGAGAGCCTCAAGGATAC3′	
*lasI F*	5′CGCACATCTGGGAACTCA3′	52 °C
*lasI R*	5′CGGCACGGATCATCATCT3′	
*lasR F*	5′CTGTGGATGCTCAAGGACTAC3′	50 °C
*lasR R*	5′AACTGGTCTTGCCGATGG 3′	
*rhlI F*	5′CTGTGGATGCTCAAGGACTAC3′	53 °C
*rhlI R*	5′AACTGGTCTTGCCGATGG3′	
*rhlR F*	5′GTAGCGGGTTTGCGGATG3′	54 °C
*rhlR R*	5′CGGCATCAGGTCTTCATCG3′	
*pqsA F*	5′GACCGGCTGTATTCGATTC3′	51 °C
*pqsA R*	5′GCTGAACCAGGGAAAGAAC3′	
*pqsR F*	5′CTGATCTGCCGGTAATTGG3′	51 °C
*pqsR R*	5′ATCGACGAGGAACTGAAGA3′	
*phzA F*	5′TGAACGGTCAGCGGTACAGGG3′	57 °C
*phzA R*	5′TCCACCGTGGCGCGATTCAG3′	
